# Structural and gene composition variation of the complete mitochondrial genome of *Mammillaria huitzilopochtli* (Cactaceae, Caryophyllales), revealed by *de novo* assembly

**DOI:** 10.1186/s12864-023-09607-8

**Published:** 2023-08-31

**Authors:** David Cruz Plancarte, Sofía Solórzano

**Affiliations:** 1 Laboratorio de Ecología Molecular y Evolución, Universidad Nacional Autónoma de México, FES Iztacala, Avenida de los Barrios 1, Los Reyes Iztacala, Tlalnepantla de Baz, 54090 Mexico; 2 Posgrado en Ciencias Biológicas, UNAM, Circuito de Posgrados, Ciudad Universitaria, Coyoacán, Ciudad de México, 04510 Mexico

**Keywords:** Cactaceae, *Mammillaria huitzilopochtli*, Mitochondrial genome, Caryophyllids

## Abstract

**Background:**

Structural descriptions of complete genomes have elucidated evolutionary processes in angiosperms. In Cactaceae (Caryophyllales), a high structural diversity of the chloroplast genome has been identified within and among genera. In this study, we assembled the first mitochondrial genome (mtDNA) for the short-globose cactus *Mammillaria huitzilopochtli*. For comparative purposes, we used the published genomes of 19 different angiosperms and the gymnosperm *Cycas taitungensis* as an external group for phylogenetic issues.

**Results:**

The mtDNA of *M. huitzilopochtli* was assembled into one linear chromosome of 2,052,004 bp, in which 65 genes were annotated. These genes account for 57,606 bp including 34 protein-coding genes (PCGs), 27 tRNAs, and three rRNAs. In the non-coding sequences, repeats were abundant, with a total of 4,550 (179,215 bp). In addition, five complete genes (*psaC* and four tRNAs) of chloroplast origin were documented. Negative selection was estimated for most (23) of the PCGs. The phylogenetic tree showed a topology consistent with previous analyses based on the chloroplast genome.

**Conclusions:**

The number and type of genes contained in the mtDNA of *M. huitzilopochtli* were similar to those reported in 19 other angiosperm species, regardless of their phylogenetic relationships. Although other Caryophyllids exhibit strong differences in structural arrangement and total size of mtDNA, these differences do not result in an increase in the typical number and types of genes found in *M. huitzilopochtli*. We concluded that the total size of mtDNA in angiosperms increases by the lengthening of the non-coding sequences rather than a significant gain of coding genes.

**Supplementary Information:**

The online version contains supplementary material available at 10.1186/s12864-023-09607-8.

## Background

In plants, mitochondria play a crucial role in providing cellular energy through respiration [1, 2], and they are also involved in various metabolic processes [3], such as stress tolerance [4] and programmed cell death [5]. In addition, some mitochondrial mutations have been associated with male sterility and they were identified in approximately 150 species, particularly in some cultivated species such as *Beta vulgaris*, *Capsicum annuum*, *Daucus carota* and *Zea mays* [6].

We recently searched the NCBI website (April 20, 2022) for complete organelle genomes of angiosperm taxa, and approximately 450 mitochondrial (mtDNA) and ~ 8000 plastidic (cpDNA) genomes were documented. This disparity in the number of sequenced genomes has led to a poorer understanding of the biology and evolution of plant mtDNA. Genomic comparisons between mtDNA and cpDNA indicate that the former is larger and more structurally complex than the latter [7]. Accordingly, mtDNA has been found to be organized either in a single molecule or multiple molecules called chromosomes, which can be arranged in linear or circular forms [8]. At present, the underlying factors and processes that determine the structural organization of plant mtDNA have not been fully elucidated. The available data suggest that in flowering plants, the number and length of mitochondrial chromosomes are not necessarily determined only by the total size of the mtDNA. For example, the parasitic mistletoe *Viscum scurruloideum* (Santalaceae) has the shortest mitochondrial genome of only 66 kbp and is organized in two chromosomes [9]. In contrast, those larger mtDNAs of *Zelkova schneideriana* with 154 kbp (Ulmaceae, MW717907) and *Corchorus capsularis* of 2 Mbp (Malvaceae, KT894204) are organized in a single chromosome. Presently, the largest mtDNA (11.3 Mbp) was documented in *Silene conica* (Caryophyllaceae), which shows a complex organization in the huge number of 128 circular chromosomes [10].

Despite this wide variation in size and structural organization, angiosperm mtDNA contains a relatively small number of genes, ranging from 28 in *Viscum scurruloideum* (Santalaceae) [9] to 69 in *Sesuvium portulacastrum* (Aizoaceae) [11]. In flowering plants, mtDNA is typically composed by three functional types of genes: protein-coding genes, tRNAs and rRNAs. As with other genomes, these functional genes are separated by non-coding DNA sequences called intergenic spacers [12]. It has been proposed that the relatively small number of genes contained in mtDNA is due to the large-scale gene migration that occurred from mitochondria to the nuclear genome along the evolutionary history of plants [13]. In fact, most of the ∼2,000 functional mitochondrial proteins currently identified are encoded in the nuclear genome, and only nearly 1% of them are encoded in mtDNA [1, 14]. In addition, gene transfer between the two cytoplasmic genomes is also common; thus, complete sequences of functional genes as well as fragments of non-coding sequences of mitochondrial origin have been identified in chloroplasts. This dynamic intergenomic gene transfer is not unusual, and it has been documented in various land plant taxa [15]. For example, the mtDNA of melon *Cucumis melo* (Cucurbitaceae) has a total size of 2.7 Mbp, and nearly 46.77% and 1.41% are from nuclear and plastidic origin, respectively [16]. Accordingly, intergenomic gene transfer is a factor that has increased the total size of mtDNA in plants [15, 17]. Additionally, in the mtDNA of angiosperms, horizontal gene transfer has been documented from different taxonomic groups, such as viruses [18], bacteria [19], fungi [20], as well as from distinct plant species [21, 22]. The mtDNA of land plants contains abundant repeated DNA sequences, most of them located at the non-coding sequences (intergenic spacers, IGS). These abundant repeats also cause substantial increases in the overall size of mtDNA [23], which could have a role in the homologous recombination and regulation of the complete replication of mtDNA [7].

Currently, the underlying factors that drive the mutation have not been fully identified for plants. However, preliminary comparisons of coding genes showed lower mutation rates in mtDNA than those estimated in plastidic (3X higher) and nuclear (16X) genomes [24, 25]. Since mutations are more constrained in coding sequences of mtDNA, they do not represent an adequate source of molecular variation for phylogenetic studies [26]. On the other hand, the widely abundant, large and continuous sequences of non-coding regions (i.e., introns and IGS) have not been explored as potential sources of molecular variation to address biological questions. Finally, plant mtDNA is likely to be imprinted with the evolutionary history of plants and may help to elucidate the enigmatic and not fully resolved evolutionary history of angiosperms.

At present, most phylogenetic studies in angiosperms have been carried out using plastidic loci (e.g., [27], [28]). However, this genome has not been effective for whole flowering groups, such as cacti species. The nearly 1,500 members of Cactaceae [29] are recognized as a monophyletic group [30]; however, their internal phylogenetic relationships have not been fully resolved (e.g., [31, 32]). In this study, we *de novo* sequenced and assembled the mitochondrial genome of *Mammillaria huitzilopochtli* D. R. Hunt. (Cactaceae, Caryophyllales). Recently, the whole cpDNA of this short-globose cactus *M. huitzilopochtli* was described [33], and its relative plastidic molecular variation was assessed [34]. The objectives of the present study were (1) to describe the structural organization of the whole mitochondrial genome in this cactus, (2) to estimate the mutation rates of coding regions among 21 species, (3) to compare our results with those reported for mtDNA from 20 other land plants, with emphasis on Caryophyllids.

## Results

### Characterization of the mitochondrial genome of *Mammillaria huitzilopochtli*

**Fig. 1:**
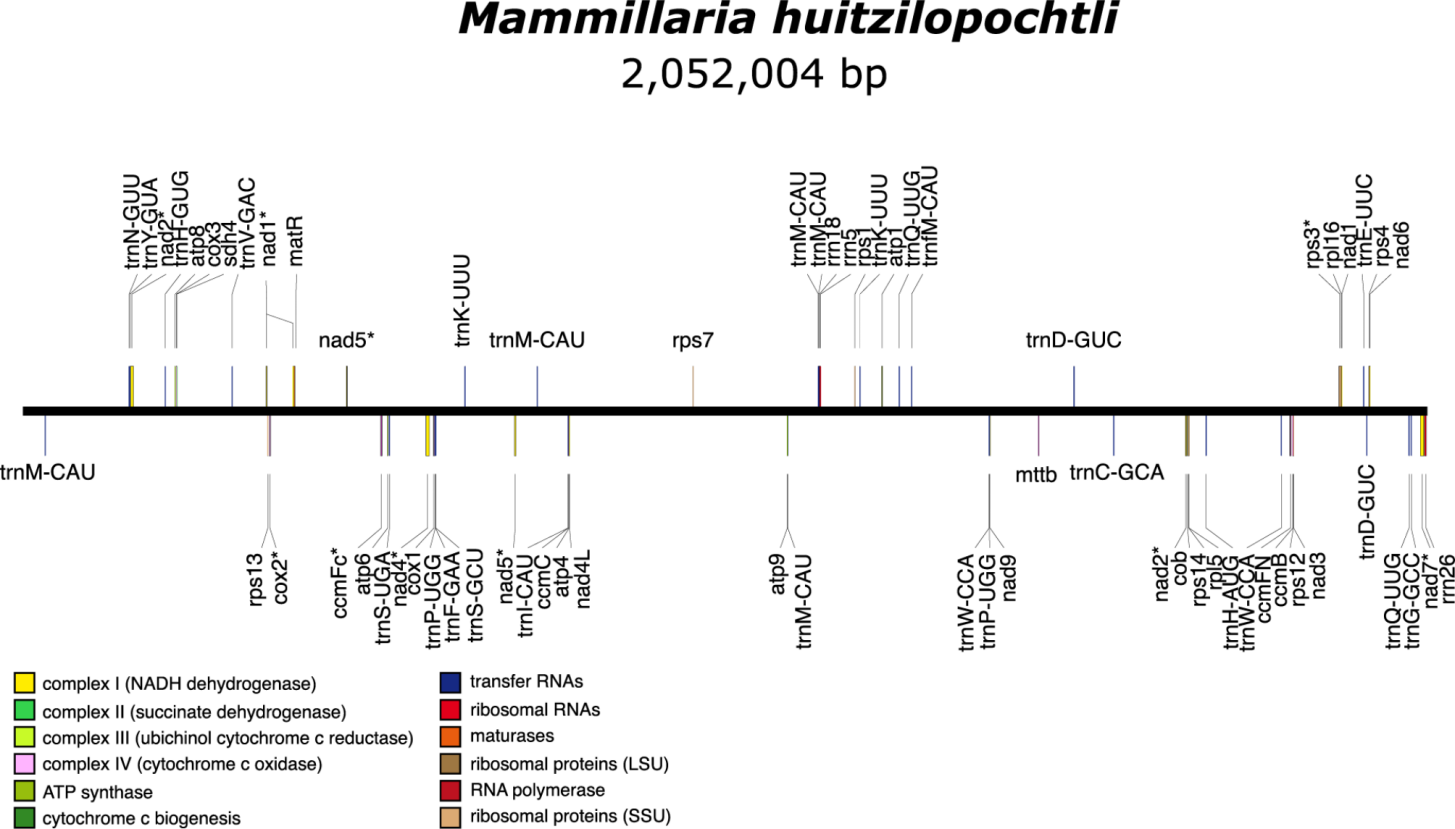
Gene composition and total size of the mitochondrial genome of *Mammillaria huitzilopochtli*. The color of the square and the label indicate the type of the protein-coding gene, excepting those indicated for tRNA and rRNAs

The newly assembled mitochondrial genome of *M. huitzilopochtli* has a total size of 2.052 Mbp and is organized in a single linear molecule. This mtDNA had a higher proportion of A’s (28.6%) and T’s (28.4%), followed by G’s and C’s (21.5% each). This genome comprised genes from 12 families: 10 of these corresponded to different types of protein-coding genes (Fig. 1).

A total of 65 distinct genes (PCGs, tRNAs and rRNAs) were annotated in the mtDNA of *M. huitzilopochtli*, six of these genes had one to four additional copies (Table 1). Thirty-four of them were protein-coding genes (PCGs), including 33 of mitochondrial origin and one (psaC) from the plastid. A total of 28 subunits of tRNAs were identified, and four of them were of plastidic origin; lastly, three subunits of rRNAs were documented (Fig. 1). The 65 annotated genes represented only 2.8% (57,606 bp) of the DNA sequence of the total genome size; consequently, 97.2% of the DNA sequences corresponded to non-coding sequences mostly located in the IGS (Fig. 1).

**Table 1:** Gene composition of the mitochondrial genome of *Mammillaria huitzilopochtli* grouped by protein coding genes, ribosomal and transfer RNAs. Protein coding genes were from ten different gene families; for each of these genes is showed its length, its start and stop codons; and the number of amino acids transcribed

Type of genes	Gene name	Length (bp)	Start codon	Stop codon	Amino acid
**I. Protein Coding Genes**					
**1. NADH dehydrogenase**	*nad1* ^a^	978	ACG	TAA	325
	*nad2* ^a^	1467	ATG	TAA	488
	*nad3*	357	ATG	TAA	118
	*nad4* ^a^	1488	ATG	TGA	495
	*nad4L*	273	ATG	TAA	90
	*nad5* ^a^	1992	ATG	TAA	663
	*nad6*	690	ATG	TAA	229
	*nad7* ^a^	1092	ATG	TAG	363
	*nad9*	579	ATG	TAA	192
**2. ATP synthase**	*atp1*	1530	ATG	TGA	509
	*atp4*	552	ATG	TAA	183
	*atp6*	726	ATG	TAG	241
	*atp8*	489	ATG	TAA	162
	*atp9*	225	ATG	CGA	74
**3. Cytochrome c biogenesis**	*ccmB*	621	ATG	TGA	206
	*ccmC*	720	ATG	TGA	239
	*ccmFC* ^a^	1341	ATG	TAG	446
	*ccmFN*	1740	ATG	TGA	579
**4. Cytochrome c oxidase**	*cox1*	1575	ATG	TAA	524
	*cox2* ^a^	834	ATG	TAG	277
	*cox3*	798	ATG	TGA	265
**5. Maturase**	*matR*	1992	ATG	TAG	663
**6. Ubiquinol cytochrome c reductase**	*cob*	1182	ATG	TGA	393
**7. Ribosomal proteins (LSU)**	*rpl5*	561	ATG	TAA	186
	*rpl16*	477	GTG	TAA	158
**8. Ribosomal proteins (SSU)**	*rps1*	606	ATG	TAA	201
	*rps3* ^a^	1686	ATG	TGA	561
	*rps4*	1098	TTG	TAA	365
	*rps7*	447	ATG	TAA	148
	*rps12*	378	ATG	TGA	125
	*rps13*	351	ATG	TGA	116
**9. Methyltransferase**	*mttb*	783	ATA	TAA	260
**10. Cytochrome b**	*sdh4*	423	ATG	TGA	140
**II. Ribosomal RNAs**					
**11. rrn**	*rrn5*	119			
	*rrn18*	1818			
	*rrn26*	2859			
**III. Transfer RNAs**					
**12. trn**	*trnC-GCA*	73			
	*trnD-GUC (1)* ^b^	75,74			
	*trnE-UUC*	72			
	*trnF-GAA*	74			
	*trnfM-CAU*	60			
	*trnG-GCC*	72			
	*trnH-AUG*	70			
	*trnH-GUG*	74			
	*trnI-CAU*	74			
	*trnK-UUU (1)*	73,73			
	*trnM-CAU (4)*	72,74,73,73,72			
	*trnN-GUU*	72			
	*trnP-UGG (1)*	75,74			
	*trnQ-UUG (1)*	72,72			
	*trnS-GCU*	88			
	*trnS-UGA*	87			
	*trnY-GUA*	83			
	*trnV-GAC* ^b^	72			
	*trnW-CCA (1)*	74,74			

Note: In parenthesis is presented the number of additional copies annotated

(a) genes with introns; (b) genes of plastidic origin

With respect to the 33 mitochondrial PCGs, 29 (87.8%) of them had the typical ATG start codon, and four had alternative codons: ACG (*nad1)*, TTG (*rps4*), ATA (*mttb*), and GTG (*rpl16*); and three types of stop codons were documented: TAA (13 PCGs), TGA (13), and TAG (6); and only the gene *atp9* had CGA. In eight genes, introns were identified that varied in number and length (Table 1): *nad7* had four introns, followed by *nad2* (3 introns), *nad4*, and *nad5* (2); and *ccmFc*, *cox2, nad1*, and *rps3* (1). The length of these introns ranged from 838 bp (*nad5*) to 2,350 bp (*nad2*). Moreover, three of these genes with introns were trans-spliced (*nad1*, *nad2*, and *nad5*), and the other five (*ccmFc, cox2, nad4*, *nad7*, and *rps3*) were cis-spliced.

With respect to the repeated sequences, a total of 1,219 microsatellites were recorded along the mtDNA of *M. huitzilopochtli*. The most abundant microsatellites were of type mononucleotide (396 repeats), followed by dinucleotide (462), trinucleotide (59), and tetranucleotide (170). In addition, 109 microsatellites showed a compound motif (i.e., two types of repeated motifs separated by a non-microsatellite sequence). Lastly, only 23 complex microsatellites that were composed of five to six nucleotides were identified, and these were distributed along the IGS (Table 2); 20 of them were abundant on the IGS of *trnD-GUC* – *cox2* (5 repeats) and *nad1* - *rps3* (4) (Table 2).

**Table 2:** Distribution and location of the microsatellites composed by five to six nucleotides. The coordinates of start and end of the microsatellite sequences refer to the assembled mitochondrial genome of *Mammillaria huitzilopochtli*

Number	(Motif) number of repeats	Start	End	Location
1	(AAGAGT)3	34,066	34,084	*trnQ*^*UUG*^ - *trnD*^*GUC*^
2	(TGAAA)3	246,455	246,470	*sdh4* - *trnV*^*GAC*^
3	(CGAAGG)5	446,131	446,161	*matR* - *trnC*^*GCA*^
4	(AAAGA)3	450,464	450,479	*matR* - *trnC*^*GCA*^
5	(CCGGG)3	596,633	596,648	*mttb* - *nad9*
6	(AGTGA)3	978,493	978,508	*atp9* - *rps7*
7	(CTCGG)3	1,037,692	1,037,707	*rps7* - *rrn18*
8	(CCTTCG)3	1,047,066	1,047,084	*rps7* - *rrn18*
9	(TTCCT)3	1,200,521	1,200,536	*rrn5* - *rps1*
10	(TCTTG)3	1,431,830	1,431,845	*nad5* - *trn*^*GCU*^
11	(ATATAT)4	1,477,325	1,477,349	*nad4* - *trnS*^*UGA*^
12	(GCCTA)3	1,604,532	1,604,547	*trnD*^*GUC*^ - *cox2*
13	(AAGGCG)3	1,645,312	1,645,330	*trnD*^*GUC*^ - *cox2*
14	(TTTTC)3	1,649,583	1,649,598	*trnD*^*GUC*^ - *cox2*
15	(GCCCA)3	1,671,137	1,671,152	*trnD*^*GUC*^ - *cox2*
16	(ACTTTC)3	1,678,787	1,678,805	*trnD*^*GUC*^ - *cox2*
17	(CTTTT)3	1,787,375	1,787,390	*nad1* - *rps3*
18	(GAGAG)3	1,801,389	1,801,404	*nad1* - *rps3*
19	(AAAAG)3	1,805,130	1,805,145	*nad1* - *rps3*
20	(CAACT)3	1,847,144	1,847,159	*nad1* - *rps3*
21	(CTAAA)3	1,963,196	1,963,211	*trnE*^*UUC*^ - *rps4*
22	(TTTCA)3	1,997,401	1,997,416	*rps4*-3’
23	(TAGAA)4	2,007,522	2,007,542	*rps4*-3’

**Fig. 2:**
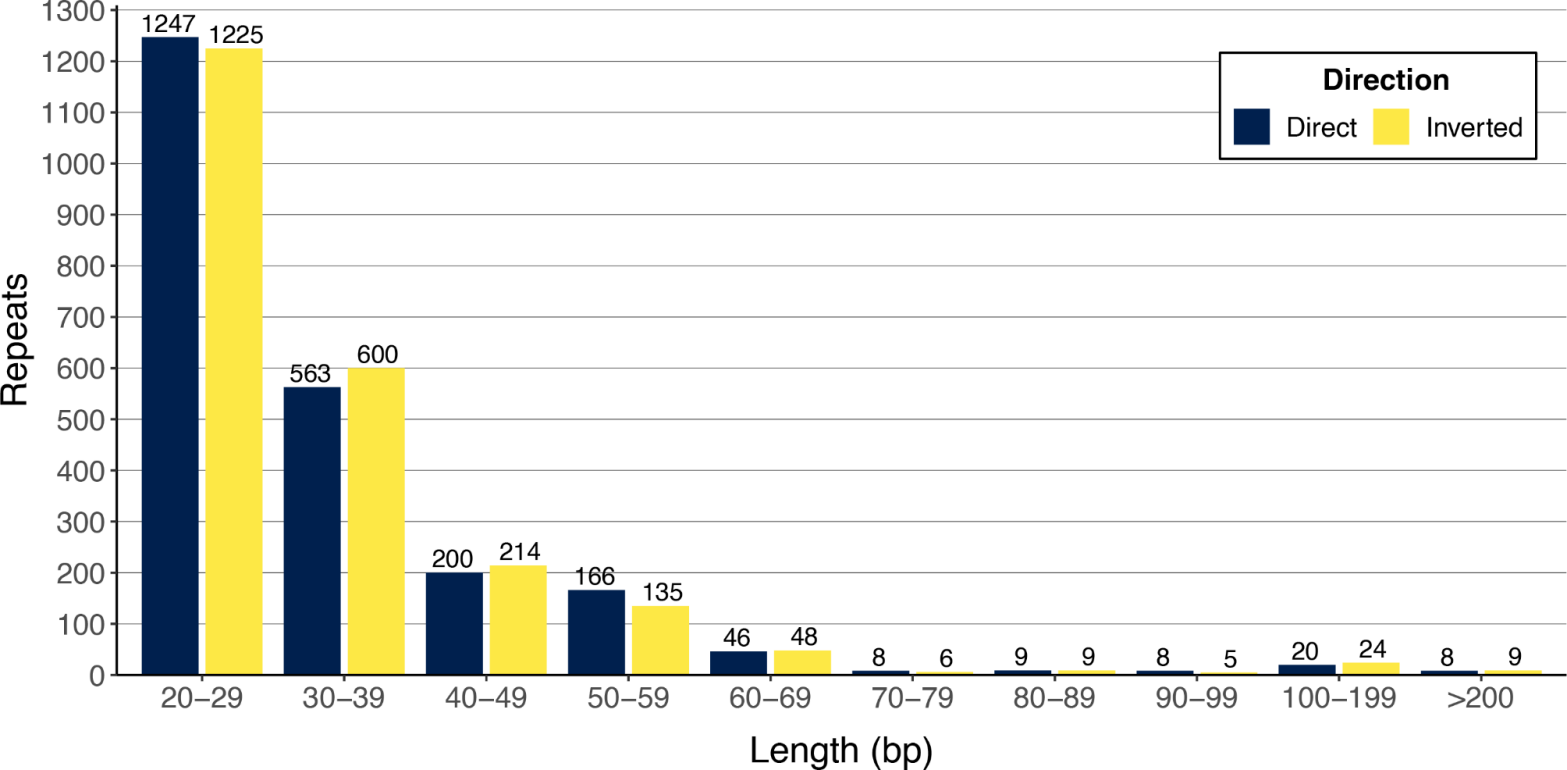
Length and direction of repeated DNA sequences documented in the mitochondrial genome of *Mammillaria huitzilopochtli*

On the other hand, direct and inverted repeats were widely and abundantly distributed across mtDNA (Fig. 2). A total of 4,550 of these repeats were documented, representing 8.73% (179,215 bp) of the total length of the genome. The most abundant repeats were the shortest ones: 20–39 bp (2,470 repeats), followed by those of 30–59 bp (1,878), 60–199 bp (183), 100–199 bp (44), and finally only 17 repeats > 200 bp were identified. Irrespective of the length, the number of repeats in direct orientation was similar to those in inverted orientation (Fig. 2).

In the mtDNA of *M. huitzilopochtli*, a total of 34 DNA sequences of plastidic origin (10,184 bp) were identified (Table 3), which were represented either by complete genes, gene fragments, or non-coding regions of the plastid. These complete copies of genes were the coding gene *psaC* (start and stop codons included) and three tRNAs: *trnD-GUC* (two copies) and one copy of *trnN-GUU* and *trnI-CAU*. The other remaining 31 DNA sequences were fragments of genes and also of IGS (Table 3).

**Table 3:** Genes, intergenic spacers (IGS) and introns of plastid origin recorded in the mitochondrial DNA of *Mammillaria huitzilopochtli.* The length, percentage of identity and coordinates obtained by comparison between genomes of mitochondria (this study), and chloroplast (MN517612). The percentage of identity, the number of mismatches and of gap opens between these two genomes

	Length (pb)	Identity (%)	Mismatches	Gap opens	Mitochondrial		Chloroplast		Gene/IGS/intron
					Start	End	Start	End	
1	976	100	0	0	1,257,330	1,258,305	92,928	93,903	*rps7* - Ψ*ndhb*
2	890	99.44	5	0	845,158	846,047	69,624	70,513	ycf2
3	848	87.38	76	11	1,916,667	1,917,495	30,751	31,586	*psaA*
4	742	90.43	71	8	1,921,118	1,921,854	10,763	11,501	*psbC*
5	731	99.32	5	0	305,474	306,204	90,124	90,854	*rrn16*
6	581	100	0	0	1,567,367	1,567,947	106,298	106,878	*atpA* - *atpF*
7	457	100	0	0	2,029455	2,029,911	3724	4180	*rpoB*
8	441	100	0	0	1,326,125	1,326,565	22,939	23,379	*rbcL*
9	421	99.29	0	0	285,799	286,219	46,334	46,754	*psbB*
10	408	91.67	34	3	1,105,343	1,105,750	65,971	66,375	ycf2
11	373	100	0	0	3	375	107,146	107,518	*atpF* (intron)
12	368	100	0	0	1,738,587	1,738,954	61,284	61,651	*matK*
13	301	98.01	6	0	97,235	97,535	92,606	92,906	*rps7*
14	264	98.86	2	1	696,708	696,972	78,457	78,719	psaC*
15	257	100	0	0	1,417,528	1,417,784	88,364	88,620	Ψycf68
16	226	96.02	8	1	839,999	840,224	94,333	94,557	Ψ*ndh* – Ψ*ycf2*
17	168	81.55	22	9	1,105,085	1,105,252	65,812	65,970	ycf2
18	164	98.17	3	0	756,462	756,625	9625	9788	*psbD*
19	160	100	0	0	306,775	306,934	88,800	88,959	Ψ*trnI*^*GAU*^ -*rrn16*
20	153	100	0	0	110,668	110,820	88,023	88,175	Ψ*trnA* - Ψ*ycf68*
21	140	95	7	0	1,231,238	1,231,377	78,948	79,087	*ndhD*
22	123	91.06	9	2	1,920,978	1,921,099	5950	6071	*trnC*^*GCA*^ - *petN*
23	122	100	0	0	1,929,365	1,929,486	85,922	86,043	*rrn23*
24	110	84.55	17	1	1,388,921	1,389,021	46,485	46,594	*psbB*
25	100	94	6	0	1,078,249	1,078,348	10,121	10,220	*psbD*
26	83	96.39	2	1	1,963,453	1,963,536	8627	8708	*trnD*^GUC^*
27	82	98.78	1	0	1,535,734	1,535,816	8627	8708	*trnD*^GUC^*
28	81	100	0	0	1,211,781	1,211,861	65,021	65,101	ycf2
29	81	93.83	5	0	264,614	264,694	105,467	105,547	*atpA*
30	78	94.87	4	0	143,568	143,645	4218	4295	*rpoB*
31	77	100	0	0	796,513	796,589	94,862	94,938	*trnI*^CAU^*
32	77	96.1	3	0	155,219	155,295	83,026	83,102	*trnN*^GUU^*
33	53	100	0	0	1,921,853	1,921,905	85,887	85,939	*rrn23*
34	48	93.75	3	0	490,750	490,797	65,638	65,685	*ycf2*

*Complete genes identified in the mitochondrial genome, Ψ indicates a pseudogene that was reported as such in chloroplast genome

### Comparison of mitochondrial DNA of *Mammillaria huitzilopochtli* to other land plants

**Fig. 3:**
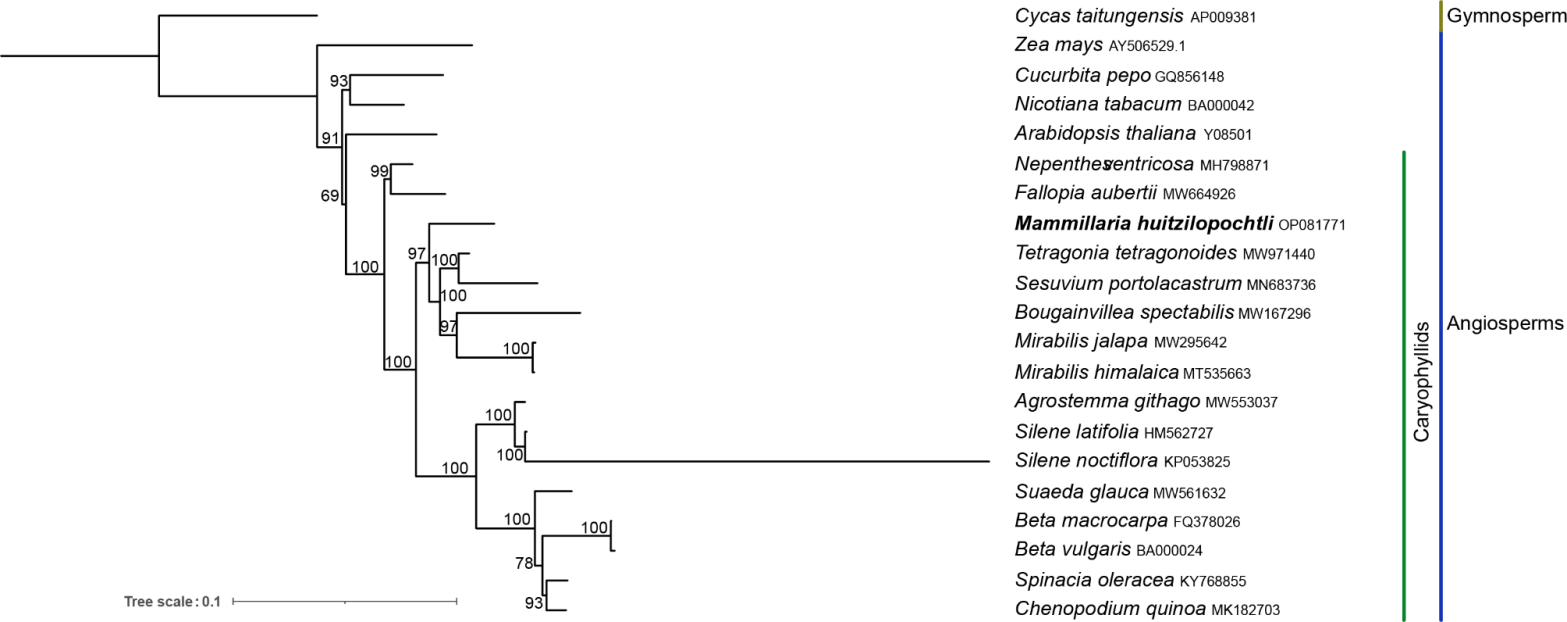
Maximum Likelihood phylogenetic tree based on 29 orthologous loci. The numbers correspond to the bootstrap percentages. The phylogenetic tree grouped the 16 Caryophyllids in a single monophyletic ingroup supported with 100% of bootstrap

The phylogenetic analysis showed a confident topology, in which the Caryophyllids were clearly grouped in a clade and had to *A. thaliana* as sister group (Fig. 3).

**Fig. 4:**
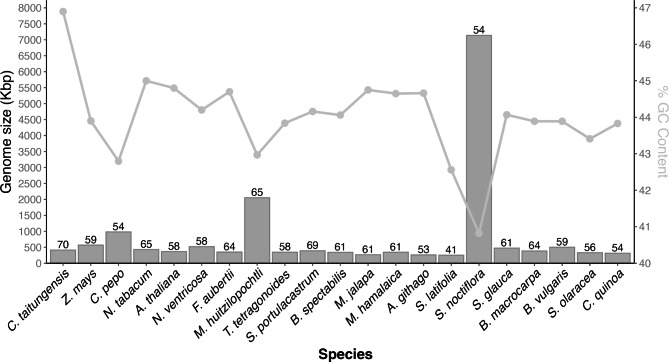
Comparison of the genome size (bars) and GC content (line) of *Mammillaria huitzilopochtli* to other 20 land plants. The number above the bar indicates the total number of genes of each genome

The comparisons carried out showed that the mtDNA of *M. huitzilopochtli* has a GC content of 42.97%, which is similar to that reported for the other 15 Caryophyllid species (Fig. 4). The average GC content in the 16 studied Caryophyllids was 43.77 ± 0.99SD. In the 21 studied plant species, there was a negative correlation between the GC content and the total length of the mitochondrial genome (r=-0.68, p = 0.00073). However, when we excluded the atypical value of *S. noctiflora*, this correlation became non-significant (r=-0.37, p = 0.11). The lowest GC content was documented in the two Caryophyllaceae species: *S. latifolia* (42.56%) and *S. noctiflora* (40.82%), with genome sizes of 235 kbp and 7.1 Mbp, respectively. Among the 21 species examined, the mtDNAs of two Caryophyllids were the largest ones: *M. huitzilopochtli* (2,052,004 bp) is the second largest genome after that of *Silene noctiflora* (Fig. 4). The average number of genes across the 21 species was 59 ± 6.34SD, and there was no correlation between their total number of genes and their total length (N = 21, r=-0.14, p = 0.56). In fact, for the largest genome of the Caryophyllid, *S. latifolia* was reported the lowest number of genes (41), whereas the gymnosperm *C. taitungensis* had the highest number of genes (70).

**Fig. 5:**
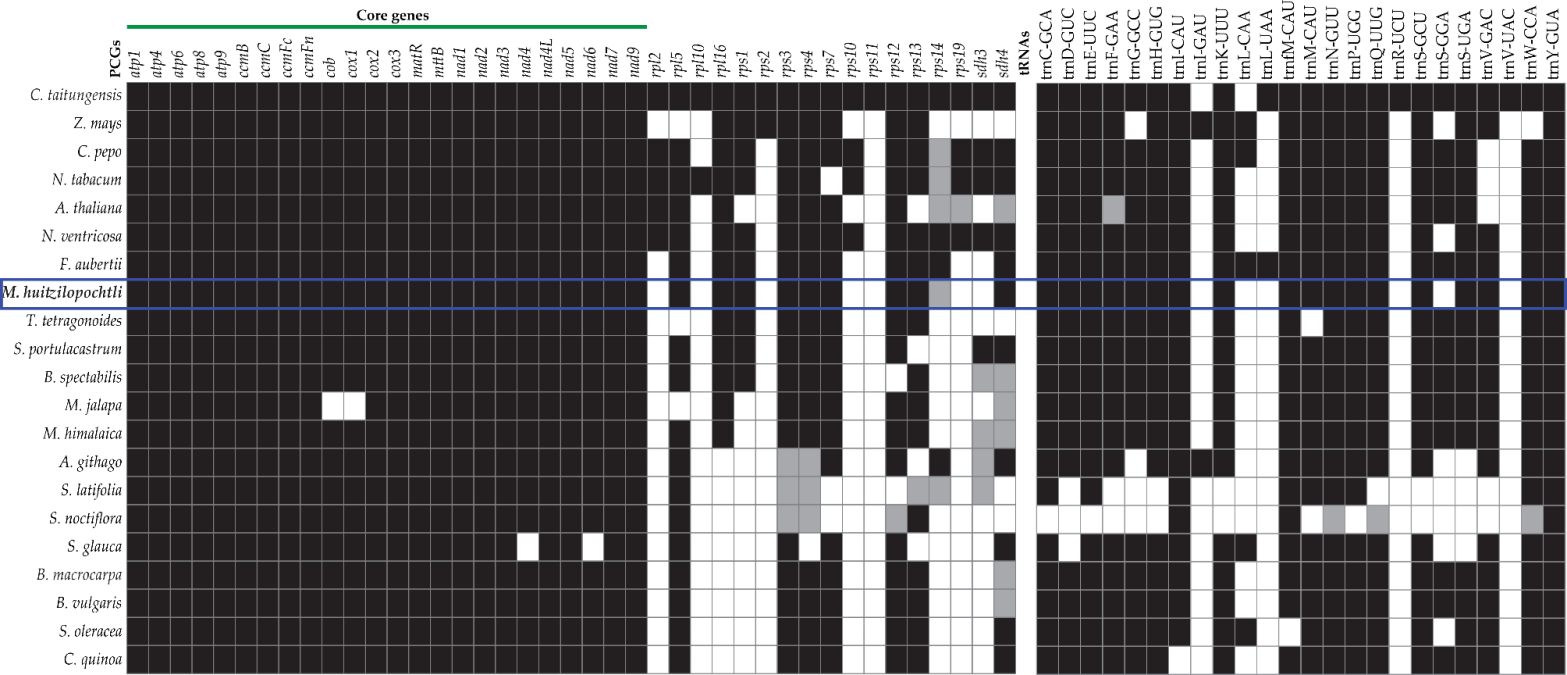
Comparison of gene content of protein coding genes and tRNAs of mitochondrial DNA of *Mammillaria huitzilopochtli* to other 20 land plant species. The color of the squares indicates if the gene was recorded (dark), absent (white), and grey (pseudogene)

With regard to the identity of the genes that composed the mitochondrial genomes, we documented that the 21 species had the three typical ribosomal units (*rrn5, rrn18* and *rrn26*) reported for land plants. However, among these species, a conspicuous variation in gene identity of PCGs was identified. The gymnosperm (*C. taitungensis*) contained the largest number of PCGs (41 genes), and the majority of angiosperms had a complete set of 24 PCGs, which are considered core genes. However, a few PCGs were missing (white squares, Fig. 5) or were incomplete sequences (pseudogenes; grey squares, Fig. 5), as was the case in *M. jalapa*, where the genes *cob* and *cox1* were absent, whereas the genes *nad4* and *nda6* were not identified in *S. glauca*. In contrast, the set of 17 genes known as variable PCGs or non-core genes was more variable across the 20 studied angiosperms. In particular, we documented the complete absence and pseudogenization of subunits of the ribosomal proteins (*rps*) and the succinate dehydrogenase (*sdh*). The cactus *M. huitzilopochtli* lacks eight of these two types of genes, and for other 12 species we identified a total of 24 pseudogenes. With respect to tRNAs, the most frequent absences were documented in *trnL-UAA* (20 species), *trnR-UCU* (20), trnV*-UAC* (20), *trnI-GAU* (19) and *trnL-CAA* (17) (Fig. 5); and pseudogenization was documented in four tRNAs but only in two species (*A. thaliana* and *S. noctiflora*). In Caryophyllids, the species *S. noctiflora* and *S. latifolia*, had a higher number of pseudogenes, 6 and 5, respectively; whereas the cactus *M. huitzilopochtli* had only one pseudogene (Ψ*rps14*; Fig. 5).

**Fig. 6:**
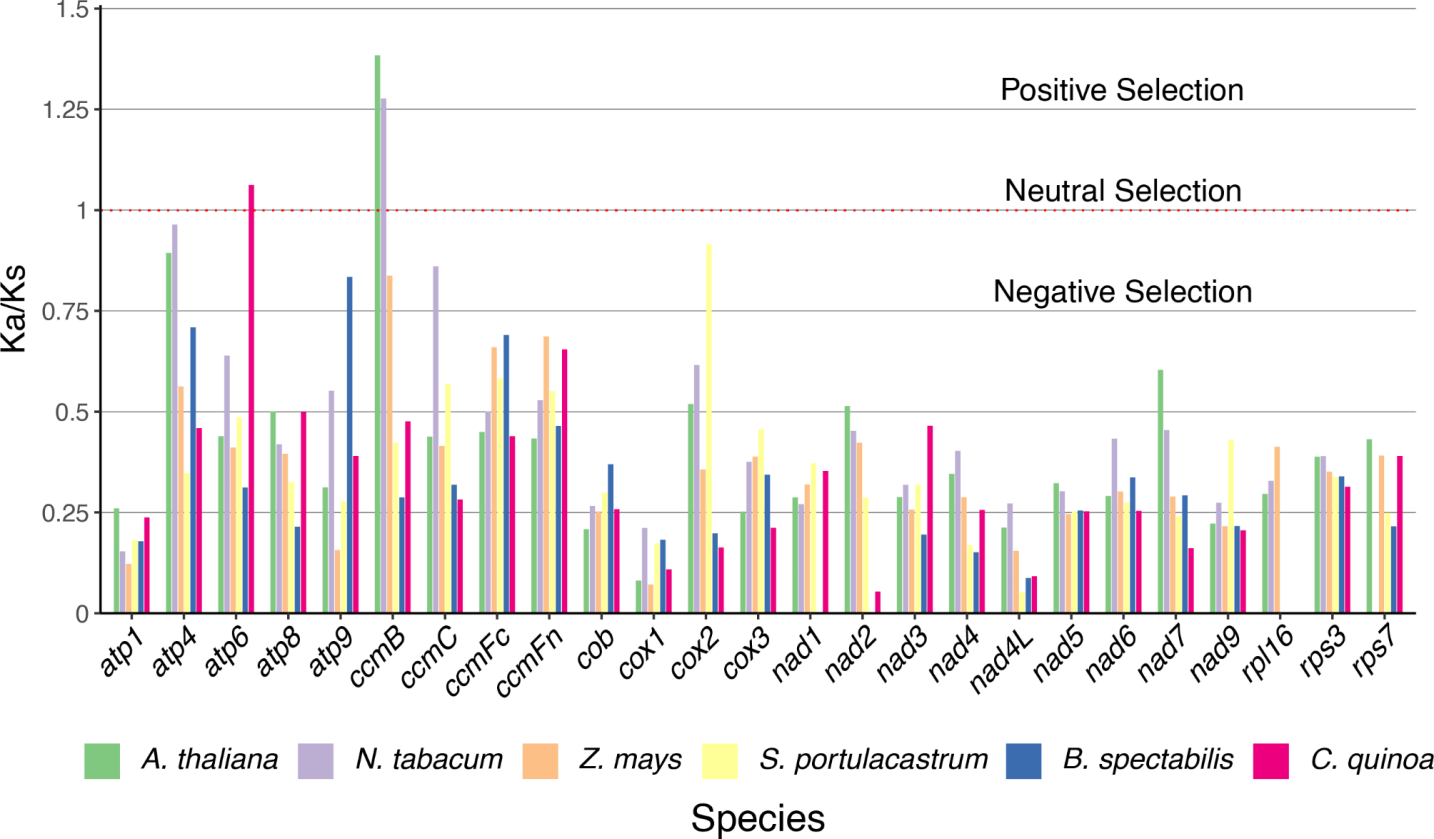
The values of Ka/Ks of 25 protein-coding genes compared between *Mammillaria huitzilopochtli* to six angiosperm species

The comparison of substitution rates in 25 genes between *M. huitzilopochtli* and six other angiosperm species (Fig. 6) showed that 23 genes had values indicating negative selection (Ka/Ks < 1, below the red horizontal line, Fig. 6). Positive selection (Ka/Ks > 1) was estimated only in the comparison of the gene *atp6* of *C. quinoa* and in *ccmB* of *A. thaliana* and *N. tabacum* (Fig. 6). No evidence for neutral selection was found.

## Discussion

This study pioneered the analysis of the complete mitochondrial genome of cactus species, and we consider that these results will open new perspectives for the phylogenetic analysis of these plants. Unfortunately, due to the lack of data, we were only able to compare our findings to other land plants that are not phylogenetically closely related; however, the comparisons focused on Caryophyllids (Amaranthaceae, Aizoaceae, Caryophyllaceae, Nepenthaceae, Nyctaginaceae, and Polygonaceae) showed similar gene content, although the strong differences in size and structural arrangement. Our findings showed that *M. huitzilopochtli* possesses the third largest mitochondrial genome (2.05 Mbp), behind the other two Caryophyllids *S. conica* (11.3 Mbp) [10] and *S. noctiflora* (7.1 Mbp) [35]. Our comparisons among 21 species suggest that total genome size does not determine: (1) structural complexity (i.e., arrangement in multiple chromosomes), (2) GC content, and (3) total number of genes, and (4) gene identity.

We identified that the variation in the total size of mtDNA among the 21 species studied was caused by the expansion and contraction of non-coding sequences, primarily by the lengthening of IGS and secondarily by introns. Thus, the total size of mtDNA expands or contracts determined by the non-coding sequences rather than by the gain/loss of coding genes. In addition, we identified that the lengthening of IGS was associated with the abundance of repeated sequences of different types, such as microsatellites, as well as direct and inverted repeats. The abundance of repeats in the IGS of land plant mtDNA is a typical observed feature [19, 36, 37], and some studies [16, 37] have suggested that IGS may receive more DNA sequences from foreign genomes. Currently, the functional role of these repeats in mtDNA has not been clearly elucidated, but it has been postulated that these repeats may participate in the replication of complete mtDNA [23]; and in repeat-mediated recombination [38, 39]; in fact, this latter process has been proposed to play an important role in the structural rearrangements of mtDNA [7, 39, 40].

Our results indicated that the mitochondrial genome of land plants tends to maintain a stable gene composition (i.e., number and types of genes), irrespective of the overall size, structural organization, and complexity in which a specific genome is arranged. We identified that the four ribosomal units and the set of 24 PCGs show a tendency to be maintained suggesting a potential key role for these genes in plants. The results suggest that phylogeny influences the number and identity of genes rather than the mtDNA’s structural features. A conspicuous result was that the gymnosperm *C. taitungensis* had the highest number of distinct genes, which is consistent with previous findings in two other conifers, *Larix sibirica* (77 genes, [41]) and *Picea sitchensis* (71, [42]); and the studied 20 angiosperms have a lower total gene number (56, this study). In these angiosperms, this drop in the number of genes was caused by the loss of different types of PCGs and tRNAs. However, we cannot confirm if these lacking genes are in the nuclear genome since it is a fact that they are not in the plastidic genome (e.g., MW894644 and MK867773). Since the set of core genes was documented in most of the 20 angiosperms, we consider that basal common evolutionary steps constrained the current gene composition in the mtDNA of flowering plants; however, this needs further verification when more complete mitochondrial genomes are available. On the other hand, the results showed that the evolutionary process of natural selection restricts mutations in the coding genes of *M. huitzilopochtli*, as indicated by the Ka/Ks values < 1 (negative selection). Consequently, coding sequences are highly conserved in this cactus, as has been recognized for most of the angiosperm species (e.g., [2, 12]).

The migration of DNA sequences of plastidic origin (complete coding genes, fragmented gene sequences and IGS) in mtDNA of the cactus *M. huitzilopochtli* has also been documented in other species [19, 37, 43]. However, the migration of complete coding genes from chloroplast to mtDNA is not common in either angiosperms [44] or gymnosperms [45]. Currently, it has not been established if these copies of plastidic origin are functional in mtDNA [17, 44]. The migration of tRNAs from chloroplasts to mitochondria is also common in land plants [43]; and in the case of *M. huitzilopochtli*, four plastidic tRNAs [33] were documented, and for these genes a functional role in the synthesis of proteins has been proposed [43]. On the other hand, the migration from the nuclear genome to mtDNA has not been extensively researched in plants, although it may occur; as was mentioned for *Cucumis melo* (Cucurbitaceae), nearly 46.47% of its mtDNA is of nuclear origin [16]. In our study, we did not evaluate sequences of nuclear origin because a complete nuclear genome for *M. huitzilopochtli* has not yet been published.

It should be noted that the primary goal of this study was not to establish the phylogenetic relationships of *M. huitzilopochtli* with other Caryophyllids due to the scarcity of complete mtDNA data available; however, the obtained phylogenetic tree revealed a concordant topology with that from previous studies based on plastidic loci [46]. Accordingly, the seven families of Caryophyllales studied here were organized according to the previously published phylogeny of 40 families belonging to this order, which was derived from 83 plastidic loci [47]. In addition, the 16 Caryophyllid species examined in this study were grouped into a monophyletic ingroup. These phylogenetic results indicate that mtDNA harbors an evolutionary history, and particularly those 29 mitochondrial loci utilized in the study have sufficient resolution to distinguish the families of the Caryophyllales order. We expect that in the future, as more complete mitochondrial genomes are published, the value of mtDNA for phylogenetic analysis will be reassessed. For instance, the recent study conducted by Rydin et al. [26] analyzed 53 species of Rubiaceae (Gentianales) based on mitochondrial and chloroplast genomes. The phylogenetic trees showed phylogenetic discordances, suggesting that future phylogenetic studies should aim to include loci from the mitochondrial, nuclear, and plastid genomes in order to study plant evolution in detail.

## Conclusions

This newly assembled and annotated complete mitochondrial genome of the cactus *M. huitzilopochtli* provides insights that will allow further comparisons with other plants, including Cactaceae. We expect that our study will contribute to elucidate biological, phylogenetic, taxonomic, and systematic issues that have not been fully resolved in Cactaceae. In the whole group of angiosperms, we consider that we are currently far from understanding the processes that drive the structural organization of mtDNA. The low mutation rates of coding genes are restricted by natural selection, which permits synonymous substitutions in DNA sequences without affecting the amino acid chains. Lastly, we encourage the sequencing of complete mitochondrial genomes in order to unravel the evolutionary puzzle of plants.

## Methods

### Genomic DNA extraction and massive sequencing

Tissue samples of *Mammillaria huitzilopochtli* D.R. Hunt were collected in 2016 from a wild population near the municipality of San Juan Bautista Cuicatlán, Oaxaca. These tissue samples were immediately stored in liquid nitrogen until experimental processing in the laboratory, where tissue samples are maintained at -80 °C for long-term genetic research.

Frozen tissue samples of 70–100 mg from a single individual of *Mammillaria huitzilopochtli* were independently processed according to the manufacturer’s instructions of the DNAeasy Plant Mini Kit (Qiagen, Germany) in order to obtain one microgram of gDNA of high molecular weight and 260/280 ≥ 1.7. This total gDNA was sent to the sequencing service provider, who prepared PE libraries with an average insert size of ~ 600 bp and sequenced in 2 × 150 cycles on TruSeq Nano DNA 350 (Illumina, USA).

### Mitochondrial genome assembly and annotation

The quality of the raw data reads was assessed using FastQC v0.11.9 [48]. Since 91.66% of the reads had Qphred ≥ 30 and no attached adapters were identified, these reads were not filtered. This whole set of reads contained three genomes; thus, we proceeded to extract only the reads of mitochondrial origin. For this, those reads of plastidic origin were mapped with BWA-0.7.17 [49], using as a reference the cpDNA published for *M. huitzilopochtli* [33]. The plastidic reads were discarded using SAMtools 1.15 [50]. The remaining reads were assembled *de novo* with NovoPlasty 4.3 [51]. The resulting assembly produced several large supercontigs (~ 10–290 kbp) that did not form a single continuous sequence. In these large supercontigs, the plant mitochondrial origin of the reads was confirmed using BLASTN [52]. All those verified mitochondrial reads were extracted directly from raw data and newly assembled using the Unicycler v.0.4.9 pipeline [53], which employs SPAdes 3.15 [54] as the assembler. This assembler was able to recover several independent and large supercontigs of approximately 300 kbp, which were visualized in the program Bandage v.0.8.1 [55]. Since short and few gaps were identified in these large supercontigs, the original raw data were used to fill in the gaps. The program Bandage identified those pairs of supercontigs that shared flanking extremes; thus, we used BBDuk [56] to search the raw data for those reads that joined each pair of flanking sequences. Successive searches with Bandage enabled us to merge all supercontigs, resulting in a single continuous linear sequence. We found that most of the original reads of mtDNA were mapped on this single linear sequence; thus, we checked uniformity with the program Integrative Genomics Viewer (IGV), which showed that the depth of coverage had an average value of 1,318X. Once the genome was completely assembled, it was fully annotated with Mitofy [17]; and all identified genes were manually curated using BLASTN [52]. The complete mitochondrial genome of *M. huitzilopochtli* assembled, annotated, and manually curated was plotted using OGDRAW [57]. This newly assembled and curated genome was characterized in terms of total size, number of chromosomes, and gene composition based on three types of genes: protein-coding genes (PCGs) that were classified according to their functional role; tRNAs and rRNAs. For each protein-coding gene, its length, start and stop codons, as well as the length of the amino acid chain transcribed, was identified. In addition, the abundant and diverse types of repeats were characterized using MISA-web [58]. We identified microsatellite type repeats (i.e., DNA sequences repeated in tandem), as well as direct and inverted repeats of at least 20 bp with REPuter [59]. Lastly, we searched for DNA sequences of plastid origin by comparing the mtDNA with the cpDNA accessed at NCBI (MN517612) previously reported [33]. This comparison was performed using BLASTN [52] with the following parameters: matching rate ≥ 70%, E-value ≤ 1e − 10, and length ≥ 40.

### Comparison of the mitochondrial genome of *Mammillaria huitzilopochtli* to other land plant species

The comparisons were carried out in detail with the other 15 Caryophyllids as well as the other four angiosperms (*Arabidopsis thaliana*, *Cucurbita pepo*, *Nicotiana tabacum*, and *Zea mays*). The gymnosperm *Cycas taitungensis* was used as an external group in the phylogenetic analysis (species evaluated are listed in Online Resource 1). The phylogenetic tree was obtained for these 21 species, and it was based on 29 orthologous loci (26,849 bp), which were identified using OrthoFinder 2.5.4 [60]. The DNA sequences of these loci comprised both coding and non-coding sequences, including IGS. The DNA sequences of these loci were concatenated and aligned with MAFFT 7.471 [61]. The best substitution model identified by ModelFinder [62] was IVM, the Maximum Likelihood analysis ran with 1000 bootstraps in IQ-TREE 1.6.12 [63], used to obtain this tree. We used this phylogenetic tree to organize the order of taxa in the comparisons made. We compared the percentage of GC content, total size, number, and identity of genes among the 21 species. We described in detail the variation in the set of genes recognized as core genes, which includes PCGs (e.g., [2, 13]) and rRNAs. We tested the statistical correlation between GC content and the total length of the 21 genomes analyzed with Pearson correlation, following the procedure described by Sokal and Rohlf [64]. In order to evaluate the relevance of natural selection on 25 PCGs of *M. huitzilopochtli*, we estimated the rate of synonymous (Ks) and no synonymous (Ka) substitutions with the other six angiosperm species (*A. thaliana*, *Bougainvillea spectabilis*, *Chenopodium quinoa*, *N. tabacum*, and *Z. mays*). These 25 PCGs were extracted from the respective complete mtDNA of each of these seven species and then aligned using MAFFT 7.471 [61]. The rate Ka/Ks was estimated with codeml [65], which was executed online on the PAL2NAL website [66]. Accordingly, the effect of natural selection was classified as negative selection if Ka/Ks < 1, positive selection if Ka/Ks > 1, and neutral selection if Ka/Ks = 1 [67].

### Electronic supplementary material

Below is the link to the electronic supplementary material.

Supplementary Material 1

Supplementary Material 2

## Data Availability

A list of the species studied and their accession IDs is provided in Table S1. The genome generated and analyzed in the current study is provided as Additional file 2. The accession number in GenBank is OP081771.

## Abbreviations

*M. huitzilopochtli*: *Mammillaria huitzilopochtli*
mtDNA: Mitochondrial DNA
cpDNA: Chloroplast DNA
PCG: Protein coding gene
IGS: Intergenic spacers
