# Supplementary material for: Structural and gene composition variation of the complete mitochondrial genome of Mammillaria huitzilopochtli (Cactaceae, Caryophyllales), revealed by de novo assembly
Source: BMC Genomics. 2023 Aug 31;24:509. doi: 10.1186/s12864-023-09607-8 (PMC10468871; doi:10.1186/s12864-023-09607-8)
Supplement: Supplementary file 1 — Supplementary Material 1 [file 12864_2023_9607_MOESM1_ESM.docx]

Table S1. Taxonomic classification of the 21 studies species alphabetically ordered. The number of the accession number of each genome deposited in the server of NCBI (https://www.ncbi.nlm.nih.gov/).

| Phylum | **Order** | **Family** | **Species** | **Accession ID** |
| --- | --- | --- | --- | --- |
| 1. Gymnospermae | 1. Cycadales | 1. Cycadaceae | *Cycas taitungensis* | AP009381.1 |
| 2. Angiospermae | 1. Brassicales | 1. Brassicaceae | *Arabidopsis thaliana* | Y08501.2 |
|  | 2. Cucurbitales | 1. Cucurbitaceae | *Cucurbita pepo* | GQ856148.1 |
|  | 3. Caryophyllales | 1. Aizoaceae | *Sesuvium portulacastrum* | MN683736.1 |
|  |  |  | *Tetragonia tetragonoides* | MW971440.1 |
|  |  | 2. Caryophyllaceae | *Agrostemma githago* | MW553037.1 |
|  |  |  | *Silene latifolia* | HM562727.1 |
|  |  |  | *S. noctiflora* | KP053825.1 |
|  |  | 3. Cactaceae | *Mammillaria huitzilopochtli* | OP081771.1 |
|  |  | 4. Chenopodiaceae | *Beta macrocarpa* | FQ378026.1 |
|  |  |  | *B. vulgaris* | BA000024.1 |
|  |  |  | *Chenopodium quinoa* | MK182703.1 |
|  |  |  | *Spinacia oleracea* | KY768855 |
|  |  |  | *Suaeda glauca* | MW561632.1 |
|  |  | 5. Nepenthaceae | *Nepenthes ventricosa* | MH798871.1 |
|  |  | 6. Nyctaginaceae | *Bougainvillea spectabilis* | MW167296.1 |
|  |  |  | *Mirabilis jalapa* | MW295642 |
|  |  |  | *M. himalaica* | MT535663.1 |
|  |  | 7. Polygonaceae | *Fallopia aubertii* | MW664926.1 |
|  | 4. Poales | 1. Poaceae | *Zea mays* | AY506529.1 |
|  | 5. Solanales | 1. Solanaceae | *Nicotiana tabacum* | BA000042.1 |
